# Identification of a novel HERV-K(HML10): comprehensive characterization and comparative analysis in non-human primates provide insights about HML10 proviruses structure and diffusion

**DOI:** 10.1186/s13100-017-0099-7

**Published:** 2017-11-02

**Authors:** Nicole Grandi, Marta Cadeddu, Maria Paola Pisano, Francesca Esposito, Jonas Blomberg, Enzo Tramontano

**Affiliations:** 1 Department of Life and Environmental Sciences, University of Cagliari, Cagliari, Italy; 2 Department of Medical Sciences, Uppsala University, Uppsala, Sweden; 3 Istituto di Ricerca Genetica e Biomedica, Consiglio Nazionale delle Ricerche (CNR), Monserrato, Cagliari, Italy

**Keywords:** Human endogenous retroviruses, Herv, HML10, Herv-k(C4), RetroTector, Cancer, Autoimmune diseases

## Abstract

**Background:**

About half of the human genome is constituted of transposable elements, including human endogenous retroviruses (HERV). HERV sequences represent the 8% of our genetic material, deriving from exogenous infections occurred millions of years ago in the germ line cells and being inherited by the offspring in a Mendelian fashion. HERV-K elements (classified as HML1–10) are among the most studied HERV groups, especially due to their possible correlation with human diseases. In particular, the HML10 group was reported to be upregulated in persistent HIV-1 infected cells as well as in tumor cells and samples, and proposed to have a role in the control of host genes expression. An individual HERV-K(HML10) member within the major histocompatibility complex C4 gene has even been studied for its possible contribution to type 1 diabetes susceptibility. Following a first characterization of the HML10 group at the genomic level, performed with the innovative software RetroTector, we have characterized in detail the 8 previously identified HML10 sequences present in the human genome, and an additional HML10 partial provirus in chromosome 1p22.2 that is reported here for the first time.

**Results:**

Using a combined approach based on RetroTector software and a traditional Genome Browser Blat search, we identified a novel HERV-K(HML10) sequence in addition to the eight previously reported in the human genome GRCh37/hg19 assembly. We fully characterized the nine HML10 sequences at the genomic level, including their classification in two types based on both structural and phylogenetic characteristics, a detailed analysis of each HML10 nucleotide sequence, the first description of the presence of an Env Rec domain in the type II HML10, the estimated time of integration of individual members and the comparative map of the HML10 proviruses in non-human primates.

**Conclusions:**

We performed an unambiguous and exhaustive analysis of the nine HML10 sequences present in GRCh37/hg19 assembly, useful to increase the knowledge of the group’s contribution to the human genome and laying the foundation for a better understanding of the potential physiological effects and the tentative correlation of these sequences with human pathogenesis.

**Electronic supplementary material:**

The online version of this article (10.1186/s13100-017-0099-7) contains supplementary material, which is available to authorized users.

## Background

The human genome is formed in small proportion by coding sequences (~2%), while it is constituted for about half of repeated elements, among which the human endogenous retroviruses (HERV) account for ~8% of it. HERVs have been acquired as the consequence of ancient retroviral infections affecting the germ line cells over several million years [1], and consequently transmitted to the offspring in a Mendelian way [2]. In the course of evolution, HERV sequences have hoarded abundant mutations, causing loss of virulence and contributing to their actual composition [3]. Despite the accumulation of substitutions, insertions and deletions, a number of HERV genes have maintained functional Open Reading Frames (ORF) and some HERV proteins are known to be involved in important physiological functions. The main examples are Syncytin-1 and -2, two Env proteins encoded by a HERV-W [4, 5] and a HERV-FRD provirus [6], respectively, providing essential fusogenic and immunosuppressive functions to human placenta [6–9]. To explain their persistence in the human genome, it has been proposed that HERVs could be neutral sequences, thus not negatively selected and removed during evolution (parasitic theory), or, conversely, they could be involved in important cellular functions leading to their positive selection over time (symbiotic theory) [10]. However, the former theory does not exclude the latter, being possible that, after the initial acquisition, the random accumulation of mutations by the viral DNA could led to the synthesis of divergent proteins that acquired a role for the host, enabling HERVs symbiotic persistence in our DNA [10, 11]. HERVs are currently divided into three main classes according to their similarity to exogenous elements: I (*Gammaretrovirus*- and *Epsilonretrovirus*-like), II (*Betaretrovirus*-like) and III (*Spumaretrovirus*-like). The further classification of HERV groups is currently based mainly on *pol* gene phylogeny, even if the taxonomy has been for a long time based on discordant criteria, such as the human tRNA complementary to the Primer Binding Site (PBS) of each group [12]. In this way, individual HERV groups have been identified based on the amino acid associated to the tRNA putatively priming the reverse transcription, i.e. tryptophan (W) for HERV-W sequences and lysine (K) for HERV-K supergroup. Among class II elements, the HERV-K sequences were originally identified due to their similarity to the Mouse Mammary Tumor Virus (MMTV, *Betaretroviruses)* [13], and are in fact classified accordingly in 10 so-called human MMTV-like clades (HML1–10) [3]. The HERV-K elements are currently highly investigated due to their possible association with human diseases, especially regarding cancer and autoimmunity. One of the most interesting HERV-K clade is the HML10 one, initially identified due to a full-length provirus integrated in anti-sense orientation within the ninth intron of the fourth component of human complement gene (*C4A*) in the class III region of the major histocompatibility complex (MHC) on chromosome 6 short arm [14]. This HML10 provirus was subsequently named HERV-K(C4), and showed a typical retroviral structure with 5′- and 3’Long Terminal Repeats (LTR) flanking *gag*, *pol* and *env* genes. The human *C4* gene is part of the so-called RCCX cassette, a genetic module composed by four genes: *STK19* (serine/threonine nuclear protein kinase), *C4* (either in an acid *C4A* form or a basic *C4B* form), *CYP21* (steroid 21-hydroxylase) and *TXN* (tenascin) [15]. Remarkably, *CYP21A2* contains a recombination site leading to the presence, in the human population, of polymorphic monomodular (69%), bimodular (17%) and trimodular (14%) RCCX cassettes, containing one, two, and three *C4* functional copies, respectively [16]. Interestingly, HERV-K(C4) presence or absence determines a dichotomous *C4* gene size polymorphism, showing a long (22,5 kb) or a short (16 kb) form, respectively [14, 17, 18]. About three quarters of *C4* genes belong to the long variant, including the HERV-K(C4) integration that could be present in 1 to 3 copies according to the *C4* harboring gene copy number. For European-diploid genome, the most common *C4* copy number is of four copies: two *C4A* and two *C4B* [16]. Subsequently, in the human genome assembly reference sequence, HERV-K(C4) provirus is present in two copies, one inserted in *C4A* and one in *C4B*, thought to be evolved from a *C4* duplication event in a non-human primate ancestor [15] and leading to the presence of two identical proviral insertions separated by ~26 Kb. Based on time of insertion calculation, HERV-K(C4) provirus integration has been estimated to be occurred between 10 and 23 million years ago (mya) [19]. Of note, MHC is the genome region being associated with more disorders than any other one, especially concerning autoimmune and infectious diseases [20].

Cell-culture studies on HERV-K(C4) expression pointed out that i) HERV-K(C4) is expressed in various human cell lines and tissues, including cells playing an important role in the immune system [18]; ii) HERV-K(C4) antisense transcripts are present in cells constitutively expressing C4, while there is no evidence of HERV-K(C4) sense transcripts [18, 21], iii) the expression of retroviral-like constructs is significantly downregulated in C4 expressing cells [21], and iv) this downregulation is dose-dependently modulated following interferon-gamma stimulation of C4 expression [18, 21]. These evidences suggested a role of HERV-K(C4) in the control of homologous genes expression through antisense inhibition as a plausible defense strategy against exogenous retroviral infections [21]. The latter could also be able to influence HML10 group expression, as shown by the enhancement of HML10 transcription in persistently (but not de novo) HIV-1 infected cells [22]. With regards to autoimmune diseases, a recent study proposed an association between HERV-K(C4) copy number and type 1 diabetes, reporting that affected individuals have significantly fewer copies of HERV-K(C4), which could be also linked to some disease-associated MHC II alleles [23]. Therefore, it has been speculated that this HML10 copy number could be a novel marker of type 1 diabetes susceptibility, and that the insertion of other HML10 elements may contribute to the protection against this disease by antisense transcripts expression [23]. However, no final proof of this has been shown yet, while a previous study analyzing the transmission of HERV-K(C4) in type-1 diabetes patients refuted its role as a potential susceptibility marker for diabetes [24], suggesting that HERV-K(C4) could just be a passive partner in human genetic reshuffling.

Overall, besides the possible role of the well studied HERV-K(C4) provirus, also other HML10 copies integrated within the human genome can be involved in the antisense control of homologous gene expression, possibly having a role in human pathogenesis. Thus, the comprehensive characterization of the HML10 group at the genomic level could provide a reliable background for understanding the specific origin, regulatory mechanisms, structure and physio-pathological effects of the transcripts reported in human cells, especially in the presence of exogenous infections, cancer and autoimmunity.

In the light of this, aiming to have a complete map of HML10 and other HERV sequences present in the human genome, we previously analyzed GRCh37/hg19 assembly, reporting a comprehensive map of 3173 conserved HERV insertions [3]. To this purpose we used the RetroTector software (ReTe), which allows the identification of full retroviral integrations through the detection of conserved retroviral motifs are their connection into chains, reconstructing the original sequence [25]. A multi-step classification approach allowed the exhaustive characterization of 39 “canonical” HERV groups, and 31 additional “non canonical” clades showing mosaicism as the consequence of recombination and secondary integrations [3]. Starting from this unique dataset, we focused on the deeper genetic analysis of individual HERV groups, which still remains a major bioinformatics goal [26], starting from the ones supposedly to be involved in human pathogenesis.

Using ReTe, we performed the first global analysis of the HML10 group presence in the human GRCh37/hg19 genome assembly, identifying a total of eight sequences that have been classified as HML10 [3]. More recently, seven of these eight HML10 elements have been further described as non-randomly distributed among chromosomes, but preferentially found nearby human genes, with a strong prevalence of intronic localization and antisense orientation with respect to the surrounding gene [27]. In the same work, three HML10 proviruses integrated in reverse orientation within human introns were investigated in cell-culture models for their promoter capacity showing, for all three, a transcriptional activity in at least one LTR [27]. Authors suggested the potential antisense negative regulation of encompassing genes that, in the case of the HML10 provirus within human pro-apoptotic DAP3 (Death-associated protein 3) gene (HML10(DAP3)), was found to be efficiently suppressed by interferon γ [27]. Interestingly, the inactivation of this HML10 provirus resulted in an increase of DAP3 expression, triggering cell death and supporting the functional relevance of these retroviral transcripts in suppressing DAP3 mediated apoptosis [27]. Considering that the HML10 group was previously reported to be expressed in various cancer cell lines [28–31], the upregulation of HML10(DAP3), as well as other HML10 proviruses, could possibly be involved in the apoptotic-resistant phenotype of human malignancies [27].

Hence, also considering that the above mentioned study [27] included a lower number of HML10 proviral elements as compared to our previously reported dataset [3], we decided to provide a complete characterization of the group at the genomic level, reporting additional information about the HML10 single members phylogeny, structure and dynamics of entry and colonization of the primate lineages, and identifying a HML10 locus not previously reported.

## Results

### Localization and characterization of HERV-K(HML10) sequences

Following the report of a duplicated HML10 integration in the C4 genes [32], in our previous analysis performed through the bioinformatics tool ReTe, a total of eight HML10 sequences were identified, seven of which were reported for the first time [3] (Table 1). Seven of these were then used in a subsequent study that did not include the HML10 provirus in locus 19p13.2 [27], possibly relying on its misleading annotation by RepeatMasker. 19p13.2 HML10 provirus, in fact, is indeed ~550 nucleotides shorter as compared to the relative annotation in Genome Browser, which improperly associated to this HML10 locus an additional 5′ portion that is albeit not part of the HML10 proviral structure, being instead an HML9 LTR (LTR14C) that probably belongs to a surrounding HML9 proviral sequence. Thus, this HML10 provirus actually lacks both LTRs and represents a secondary proviral insertion separating a pre-existent HML9 provirus 5’LTR (flanking the HML10 provirus in 5′) from the rest of its internal sequence (flanking the HML10 provirus in 3′).

**Table 1:** HML10 proviral sequences localized in the human genome GRCh37/hg19 assembly

Locus	Coordinates ^a^	Length	First reference	RVNR ^b^	Genomic context	Secondary integrations
1p36.13	1:20,253,380–20,259,203 (−)	5824	Vargiu 2016	5836	intergenic	–
1p22.2	1:89,551,973–89,554,309	2337	this study	–	intergenic	–
1q22	1:155,661,620–155,669,312 (−)	7693	Vargiu 2016	6073	DAP3 (+) L1 MB7 (−)	AluSp 155,663,467–155,663,784 (+) MER11 155,667,171–155,668,256 (−)
6p22.1	6:27,155,300–27,164,058 (+)	8759	Vargiu 2016	2101	intergenic	AluY 27,158,573–27,158,903 (+) AluYc 27,158,904–27,159,195 (+) AluY 27,159,341–27,159,663 (+) AluY 27,159,784–27,160,001 (−) LTR13A 27,162,010–27,163,209 (−)
6p21.33	a) 6:31,952,469–31,958,829 (−)	6361	Tassabehji 1994	2116	C4A (+)	–
	b) 6:31,985,207–31,991,567 (−)	6361	Tassabehji 1994	2115	C4B (+)	–
6q22.31	6:122,825,990–122,833,238 (−)	7249	Vargiu 2016	2320	PKIB (+)	AluY 122,827,840–122,828,145 (−) AluY 122,829,905–122,830,202 (−) AluY 122,830,590–122,830,893 (−)
19p13.2	19:7,860,947–7,865,932 (−)	4986	Vargiu 2016	4599	intergenic	AluY 7,861,800–7,862,107 (−) AluY 7,862,886–7,863,179 (+) AluY 7,863,787–7,864,090 (−) AluY 7,865,512–7,865,832 (+)
19q13.41	19:52,964,148–52,969,750 (−)	5458	Vargiu 2016	4762	ZNF578 (+)	–
Yq11.221	Y:15,105,784–15,113,006 (−)	7223	Vargiu 2016	5104	L1M3f (−)	LTR2B 15,106,449–15,106,924 (−) AluY 15,111,205–15,111,507 (−)

^a^Chromosome: start-end (strand). Positions are referred to the human genome sequence, assembly GRCh37/hg19

^b^Individual sequences identifiers in the first reference study (Vargiu et al. 2016, [3])

Regarding the previous identification of HML10 genomic loci, it should be considered that ReTe uses a collection of generic conserved motifs for HERV sequences recognition, which can be mutated or lost in defective proviruses [3], possibly constituting a “bias” responsible for the missed detection of less conserved HERV group members. Hence, as previously described for the HERV-W group [33], to complete the HML10 sequences identification the human genome we also performed a traditional BLAT search in Genome Browser using the RepBase HERV-K(C4) provirus reference sequence (assembled as LTR14-HERVKC4-LTR14) [34] as a query. This approach confirmed the presence of the eight HML10 proviruses previously identified by ReTe [3] and revealed the presence of an additional HML10 provirus in locus 1p22.2, with an overall number of nine HERV-K(HML10) sequences in the human genome (Table 1).

**Fig. 1:**
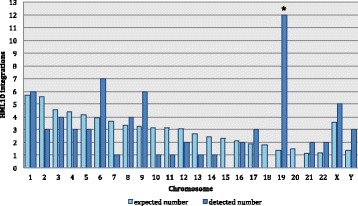
Chromosomal distribution of HML10 proviruses and solitary LTRs. The number of HML10 elements integrated in each human chromosome is depicted and compared with respect to the number of expected random insertion events based on chromosomal length. To have a more reliable estimation, we considered the number of proviruses identified by Vargiu et al. 2016 [3] as well as the solitary LTR relics, as reported by Broecker et al. 2016 [27], also representing previous integration events. The two sequences in locus 6p21.33, being a duplication of the same proviral integration, were counted as a single provirus. * statistically significant based on chi-square test (*p* < 0,0001)

In agreement with the previously adopted nomenclature [35], we indicated the HML10 sequences using their unique chromosomal position and, if more sequences were present in the same locus, we used consecutive letters (“a” and “b”) to univocally indicate each of them (Table 1). Overall, HML10 proviral sequences were present in chromosomes 1, 6, 19 and Y. Particularly, chromosome 6 held 3 integrations (including the duplicated proviral sequence in locus 6p21.33), chromosomes 1 and 19 showed 3 and 2 sequences, respectively, and 1 element was found in chromosome Y. The number of HML10 elements found in each chromosome, including the previously reported solitary LTR relics [27], was compared to the expected number of integrations based on the single chromosomes size (Fig. 1), considering that the current solitary LTRs are ancestral proviral insertions that underwent LTR-LTR homologous recombination. Results showed that the number of HML10 integration events observed is often discordant with respect to the expected amounts, suggesting a non-randomly integration pattern of the group in the various chromosomes. In particular, most of human chromosomes showed a number of HML10 insertions lower than expected, with the exception of chromosomes 6, 9, 17, 21, 22, X and Y that held around twice the number of expected insertions, reaching a 9-fold increase in chromosome 19. For some of these chromosomes, such as 17 and 19 ones, an enrichment in HML10 insertions could be expected considering their particularly high gene density, as the HML10 proviruses are known to show prevalent integration in intronic regions [3, 27], as observed also for other HERV groups preferentially inserted in proximity to human genes [36]. In chromosomes with low recombination rate, such as chromosome Y, the relative abundance of HERV may instead be due to the absence of major recent rearrangements [36], or to an higher rate of HERV fixation in the male germ line, favoring HERV persistence [37]. To verify the non-randomness of HML10 integrations distribution in human chromosomes, we compared the actual number of HML10 loci with the expected one with a random integration pattern through a chi-square (*χ* ^2^) test. Results rejected the null hypothesis that HML10 sequences are randomly distributed in the human genome, supporting an overall non-random integration pattern through an highly significant *p* value (*p* < 0,0001). However, when applied to the individual chromosomes, the same test showed that the variation between observed and expected number of HML10 integration was not statistically significant (mean p value = 0,4) except for chromosome 19, which was confirmed to be significantly enriched in HML10 sequences (p < 0,0001) making hence the overall statistics significant (Fig. 1).

**Fig. 2:**
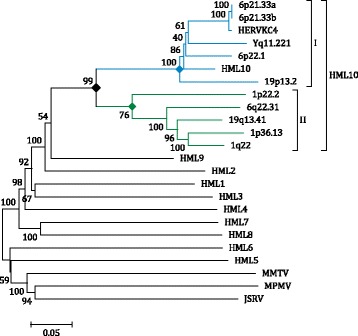
Phylogenetic analysis of the full-length retrieved sequences and other endogenous and exogenous Betaretroviruses. The main HML10 phylogenetic group is indicated. The two intragroup clusters (I and II) are also annotated and depicted with blue and green lines, respectively. Evolutionary relationships were inferred by using the Neighbor Joining method and the Kimura-2-parameter model. The resulting phylogeny was tested by using the Bootstrap method with 1000 replicates. Length of branches indicates the number of substitutions per site

In order to confirm the belonging of the newly identified sequence to the HML10 group, we performed a Neighbor Joining (NJ) phylogenetic analysis of the full-length proviruses, including the HML1–10 RepBase reference sequences [34] assembled as LTR-internal portion-LTR from Dfam database [38] as well as the main representative exogenous Betaretroviruses (MMTV; Mason-Pfizer Monkey Virus, MPMV and Jaagsiekte sheep retrovirus, JSRV) (Fig. 2). The phylogenetic analysis confirmed that the newly identified partial proviral sequence in locus 1p22.2 belongs to the HML10 group, clustering with the previously identified HML10 elements and with the Dfam and RepBase HML10 HERV-K(C4) proviral reference sequences with a 99 bootstrap support. Overall, this phylogenetic group is clearly separated from the other endogenous and exogenous Betaretroviruses, even if sharing higher similarity with the HML9 and HML2 references. Interestingly, within this main phylogenetic group we observed two different clusters, that we named type I and II, which were  statistically supported by bootstrap values (100 and 76, respectively) (Fig. 2). Type I HML10 sequences (blue lines) include both the Dfam HML10 reference and the HERV-K(C4) representative provirus, corresponding to the duplicated integrations in locus 6p21.33. Type II elements (green lines) showed a more divergent structure with respect to the group references, especially regarding the proviral locus 1p22.2 that is also less related to the other cluster II members.

### HML10 proviruses structural characterization

**Fig. 3:**
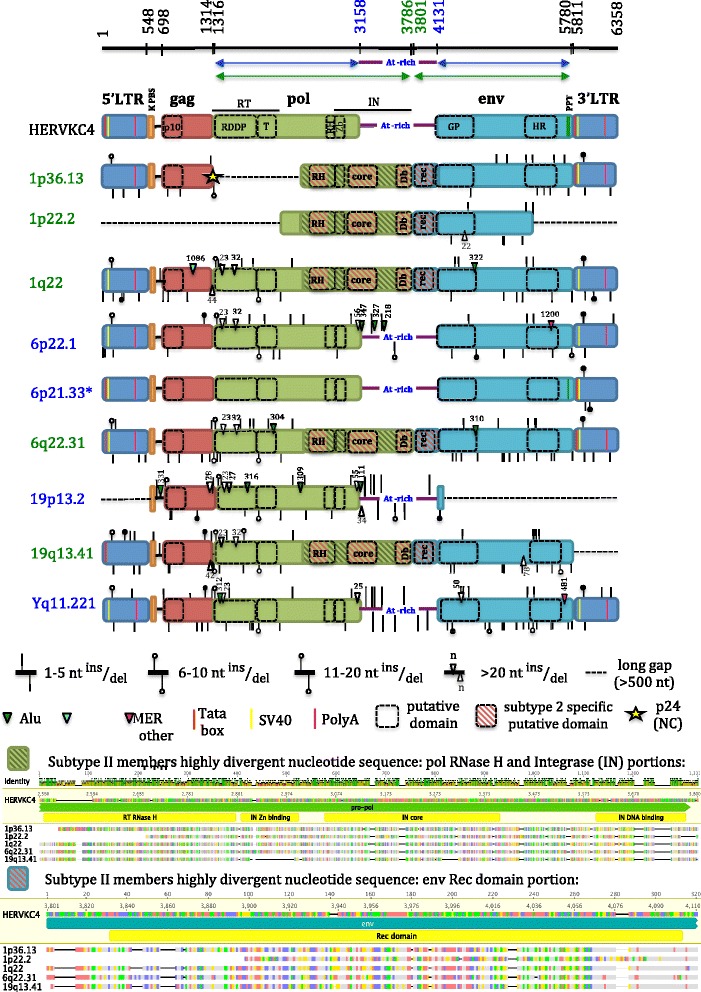
HML10 proviruses structural characterization. Each HML10 provirus nucleotide sequence has been compared to the reference sequence HERV-K(C4) (RepBase). Nucleotides insertions and deletions, LTR regulatory elements and retroviral genes predicted functional domains are annotated. Type II proviruses are reported in red and showed a more divergent nucleotide sequence, especially in *pol* RNase H and IN portions and *env* 5′ region (red stripes). Due to the high number of nucleotide changes, the comparison of these portions to the reference is depicted separately. RT: Reverse Transcriptase; RDDP: RNA dependent DNA polymerase; T: thumb; RH: Ribonuclease H; IN: Integrase; Zb: Zinc binding; Db: DNA binding; GP: glycoprotein; HR: Heptad Repeats. Type I proviruses present in the correspondent portion an A/T-rich stretch previously reported for HERV-K(C4) between *pol* and *env* genic regions

Considering that the phylogeny of the HML10 full-length proviruses revealed the clear presence of type I and II sequences, we analyzed in detail the nucleotide structure of the individual members to gain a comprehensive knowledge of the uniqueness of each HML10 locus and to characterize the main differences between the two types. To this aim, we aligned all the HML10 proviruses nucleotide sequences to the RepBase reference LTR14-HERVKC4-LTR14, namely HERV-K(C4), corresponding to the two duplicated proviral insertions in locus 6p21.33. For each HML10 provirus, we annotated all insertions and deletions up to 1 nucleotide as well as the presence of the main structural and regulatory features, as referred to the LTR14-HERVKC4-LTR14 RepBase sequence (Fig. 3). Particularly, we verified the conservation of LTR motifs relevant for retroviral expression, i.e. a Tata box (TATAAA, nucleotides 30–35 and 5840–5845), a SV40 enhancer (GTGGAAAG, nucleotides 65–72 and 5875–5882) and a PolyA signal (AATAAA, nucleotides 384–389 and 6194–6199), as well as the conservation of the PBS sequence (nucleotides 552–569) and the polypurine tract (PPT, nucleotides 5786–5798). We also analyzed the presence of functional domains in the retroviral genes, as predicted by the NCBI tool for conserved domains search [39] (Fig. 3). In addition, we assessed whether the ~830 nucleotides A/T-rich stretch previously reported between the *pol* and *env* genes of HERV-K(C4) proviral insertion (from nucleotide 3159 to nucleotide 3189) [14] was present in any other HML10 sequence. Interestingly, a correspondent portion with a comparable enrichment in A/T nucleotides (ranging from about 67% to 73%) was identified in type I proviruses only, being present also in all the members other than HERV-K(C4) (data not shown). Overall, the HML10 proviruses showed a complete retroviral structure, and the analysis allowed us to better define the location of the main retroviral genes with respect to what has been previously reported in RepBase database (Fig. 3). The majority of HML10 proviruses retained two LTRs (nucleotides 1–548 and 5811–6358) flanking the *gag* (698–1314), *pol* (1316–3786) and *env* (3801–5780) genes. Some HML10 proviral sequences, however, were defective for at least one retroviral element: loci 1p22.2 and 19p13.2 lack, for example, both LTRs, a portion of the *env* gene and, in the case of 1p22.2, the PBS sequence and the whole *gag* gene. Locus 19q13.41 lacks the 3’LTR, while locus 1p36.13 lacks the 5’portion of *pol* gene but, remarkably, it present indeed the *gag* p24 nucleocapsid region, which resulted instead absent in all the other analyzed sequences. Regarding the LTR regulatory sites (Tata box, SV40 and PolyA), all the HML10 proviruses LTRs showed nucleotide changes in at least one motif, except for locus 6q22.31 that showed conserved nucleotide sequences for all the considered features in both LTRs, in line with its reported promoter activity in cell cultures [27] (Fig. 3). Moreover, the presence of the above-mentioned A/T-rich stretch in type I HML10 sequences constitutes a variation in the *pol* and *env* genic structure, because this portion has traditionally been considered as not included in the sequence of these two genes in HERV-K(C4) [14] and, actually, its presence in type I sequences corresponds to the absence of any putative Pol and Env functional domains. Thus, while the *pol* gene start position and the *env* gene terminal position are common to both types members, type I *pol* and *env* genes appear to end before (*pol*, nucleotide 3158), and start after (*env*, nucleotide 4131), the correspondent genes in type II HML10 sequences, respectively (Fig. 3). The NCBI search for conserved domains predicted the presence of some functional features shared by all the group members retaining the harboring gene portion: a Gag p10 domain (core region), Pol Reverse Transcriptase (RT) RNA Dependent DNA Polymerase (RDDP) and thumb domains, a Pol Integrase (IN) Zinc binding site, and Env Glycoprotein and Heptad Repeats regions. None of the HML10 elements retained instead any domain that could suggest the presence of a *pro* gene, which seems to be defective for the whole group. In addition, it is interesting to note that some other predicted domains were identified only in a subset of HML10 elements, all belonging to type II sequences (Fig. 3). The latter showed, in fact, a highly divergent nucleotide structure when compared to the HERV-K(C4) reference, in *pol* Ribonuclease H (RNase H) and IN portions, as well as in the 5′ region of *env* gene. Of note, these peculiar genic regions of type II proviral sequences correspond, in sequence positions, to the above-mentioned A/T-rich stretch found exclusively for HML10 type I elements, further confirming the high nucleotide divergence of such element with respect to the type II *pol* 3′ and *env* 5′ portions (Fig. 3). The search for conserved motifs in such regions revealed the peculiar presence, in type II HML sequences, of i) a longer putative Pol RNase H domain; ii) an IN core domain, iii) an IN DNA binding site and iv) an Env Rec domain, which were contrarily not found in any of the HML10 type I proviruses. Particularly, the presence of a putative Rec domain was unexpected, since such accessory protein has been reported to be present in the HERV-K(HML2) proviruses only [40–42], where its expression has been tentatively linked to cancer development. Thus, we characterized in more detail such HML10 Rec domain through the bioinformatics analysis of the correspondent putative proteins and their comparison to the already characterized HML2 Rec proteins present in UniProt database [43].

### Characteristics of the newly identified HML10 Rec putative proteins

**Fig. 4:**
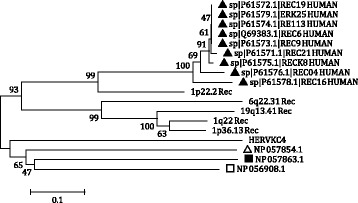
Phylogenetic analysis of the HML10 subtype II Rec putative proteins. The HML10 subtype II proviruses nucleotide sequences corresponding to a predicted Rec domain were translated and the obtained putative proteins (puteins) were analyzed in a NJ tree including previously reported HERV-K HML2 Rec proteins (black triangles) and the analogues HIV-1 Rev. (white triangle), HTLV-1 Rex (black square) and STLV Rex (white square) proteins. Evolutionary relationships were inferred by using the Neighbor Joining method and the p-distance model. The resulting phylogeny was tested by using the Bootstrap method with 1000 replicates. Length of branches indicates the number of substitutions per site

In order to characterize in more detail the Rec coding region in HML10 subtype II elements, we built a NJ phylogenetic tree of the five subtype II proviruses Rec sequences after their bioinformatics translation in the correspondent putative proteins (puteins) (Fig. 4). The amino acids sequences of nine previously published HERV-K(HML2) Rec proteins as well as the analogues Human Immunodeficiency Virus 1 (HIV-1) Rev and Human T Lymphotropic Virus 1 (HTLV-1) and Simian T Lymphotropic Virus 1 (STLV-1) Rex proteins were included as references (see Methods). As shown in Fig. 4, 1p22.2 Rec putein showed the highest relation to the HERV-K(HML2) Rec proteins, with a 99 bootstrap value. This cluster was itself related to the other four HML10 Rec puteins, supported by a 93 bootstrap value. Differently, the putein obtained from the translation of the correspondent nucleotide portion of HERV-K(C4), used as representative for type I HML10 elements, did not show remarkable phylogenetic similarity to any Rec sequence, as suggested by the presence of the A/T-rich stretch in this region.

**Fig. 5:**
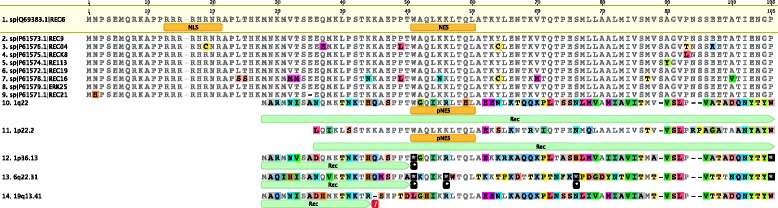
Structural comparison between HERV-K HML2 Rec proteins and the putative HML10 Rec amino acid sequences. The HML10 subtype II proviruses nucleotide sequences corresponding to a predicted Rec domain were translated and the obtained putative proteins (sequences 10–14) were compared to the HERV-K HML2 Rec proteins reported in UniProt (sequences 1–9). Coloured residues represent amino acid substitutions with respect to Q69383 HML2 Rec protein reference sequence. The presence of stop codons is indicated with a star into a black square, the occurrence of frameshifts is indicated with a red square. The putative protein theoretically originated by the inferred ORFs are indicated with a light green arrow. The localization of HML2 Rec proteins Nuclear Localization Signal (NLS) and Nuclear Export Signal (NES) as well as the correspondent putative signals in HML10 Rec puteins are also indicated

To further investigate the possible relevance of the five Rec puteins identified in type II HML10 sequences, we analyzed the occurrence of premature internal stop codons and frameshifts as compared to UniProt HML2 Rec proteins (Fig. 5). Remarkably, two of the five HML10 Rec ORFs (locus 1q22 and 1p22.2) showed an intact structure devoid of premature stop codons and frameshifts, theoretically encoding for 76 and 72 amino acids puteins, respectively (Fig. 5). 1p36.13 Rec putein showed instead a single internal stop codon at residue 24, whose reversion could theoretically lead to the production of a full-length putein. The Rec puteins in HML10 loci 6q22.31 and 19q13.41 show a more defective structure, being affected by 3 premature stop codons (6q22.31, positions 24, 29 and 49) and one internal frameshift (19q13.41, between residues 17 and 18), respectively. Thus, we focused our attention on the two HML10 Rec puteins with potentially intact ORFs (locus 1q22 and 1p22.2), evaluating the preservation of important functional domains as described for HERV-K(HML2) Rec proteins (Fig. 5). The latter present, in fact, two motifs needed for nuclear localization and export (NLS and NES, respectively) [44]. The analysis showed that, while all HML10 Rec puteins apparently lack the NLS portion, both 1q22 and 1p22.2 Rec puteins present a recognizable putative NES domain (Fig. 5).

### Estimated time of integration

**Fig. 6:**
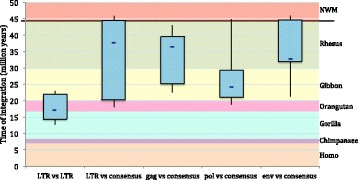
Overview of HML10 group colonization of primate lineages. Boxplot representations of HML10 group period of entry in primate lineages. The estimated age (in million years) was calculated considering the divergence values between i) the 5′ and 3′ LTRs of the same provirus; ii) each LTR and a generated consensus; iii) *gag*, *pol* and *env* genes and a generated consensus. The approximate period of evolutionarily separation of the different primate species are also indicated and have been retrieved from Steiper et al. 2006 [70] and Perelman et al. 2011 [71]. Boxes represent the main period of HML10 group diffusion in primates based on the different approaches of calculation, including from 25 to 75 percentiles and showing the mean value as a blue dash. Whiskers indicate the minimum and maximum estimated age

A special property of proviral sequences is that their LTRs are identical at the time of integration, so that their divergence (D) after endogenization depends on the genome random mutation rate per million years, allowing to estimate the time of integration (T) of each provirus [45]. Even if this method has been widely used to calculate the HERV sequences approximate age, it is affected by important limitations, as previously reported [33]. Firstly, it is not applicable to those proviruses lacking one or both LTRs and, secondly, it may underestimate T values, as it has been shown comparing the T values to the presence in non human primates of the HERV proviruses orthologous sequences [33]. For these reasons, we estimated the HML10 proviruses age through a multiple approach of T calculation, based on the D percentage value between i) the 5′ and 3′ LTRs of the same provirus (LTR vs LTR, possible for 7/9 HML10 sequences); ii) each LTR and a generated LTR consensus sequence; and iii) the *gag*, *pol* and *env* genes and a generated consensus sequence. Both consensus sequences have been generated following the majority-rule by the multiple alignments of all HML10 proviruses. Briefly, for each approach, the T value has been estimated by the relation T = D%/0,2%, where 0,2% represents the human genome random mutation rate expressed in substitutions/nucleotide/million years [46–48]. With regards to the D between the two LTRs of the same provirus, the obtained T value has been further divided for a factor of 2, considering that after endogenization each LTR accumulates random substitutions independently. For each provirus, the final T value has been calculated as the average of the T values obtained with the different approaches. Noteworthy, the final T value has also been validated by the identification of the Oldest Common Ancestor (O.C.A., i.e. the most distantly related primate species presenting the correspondent orthologous insertion), which also provides details on the period of proviruses formation (Table 2 and Fig. 6).

**Table 2:** HML10 sequences estimated time of integration

	LTR vs LTR	LTR vs consensus	*gag* vs consensus	*pol* vs consensus ^a^	*env* vs consensus ^b^	*Average*	O.C.A. ^c^
1p36.13	14.1	21.0	22.5	no *pol* (62 nt only)	31.9	*22.4*	rhesus
1p22.2	no 5′ and 3’LTRs	no 5′ and 3’LTRs	no *gag*	no *pol*	45.0	*45.0*	rhesus
1q22	14.7	44.1	35.7	28.9	32.7	*31.2*	rhesus
6p22.1	12.7	36.5	43.0	18.9	32.8	*28.8*	rhesus
6p21.33a	22.9	18.0	25.2	21.3	21.3	*21.7*	rhesus^d^
6p21.33b	22.9	18.0	25.2	21.3	21.3	*21.7*	orangutan^d^
6q22.31	17.2	38.8	38.9	44.8	35.1	*35.0*	rhesus
19p13.2	no 5′ and 3’LTRs	no 5′ and 3’LTRs	^e^	20.8	no *env* (48 nt only)	*20.8*	rhesus
19q13.41	no 3’LTR	46.0	37.4	27.2	45.9	*39.1*	rhesus
Yq11.221	20.8	45.2	41.5	30.4	44.7	*36.5*	rhesus
*Average*	*17.9*	*33.5*	*33.7*	*26.7*	*34.5*	*28,58*	

^a^partial sequence: nucleotides 1277–2571 in LTR14-HERVKC4-LTR14

^b^partial sequence: nucleotides 4103–5810 in LTR14-HERVKC4-LTR14

^c^Oldest Common Ancestor

^d^Provirus loss in various intermediate species: chimpanzee, gorilla, orangutan and gibbon (6p21.33a); chimpanzee, gorilla, gibbon and rhesus (6p21.33b)

^e^sequence showing an highly divergent gag sequence, giving an estimated T of 165,7 that was not taken into account for the final T calculation

In general, the HML10 group spreading in the primate lineages occurred between 40 and 20 mya, after the divergence between New World Monkeys and Old World Monkeys, with the majority of proviral insertions occurring in Rhesus macaque (Table 2 and Fig. 6). It is interesting to note that, as previously observed [33], the LTR vs LTR method gave significantly lower T values than the consensus based approaches (*p* < 0,001), showing, in fact, a D value average of 3,6% versus the 6% D average obtained with the consensus based methods. Thus, it can be concluded that T values obtained with the sole traditional LTR vs LTR approach could generally led to some underestimation, possibly indicating an earlier integration period instead of the actual one, which was also confirmed by the proviruses O.C.A.. A similar underestimation, even if with lower confidence (p < 0,05), was observed in the genes vs consensus method when comparing the T value calculated with the *pol* gene to the ones calculated for the *gag* and *env* genes, possibly suggesting a lower variability of the *pol* region, that is in fact known to be generally the most conserved retroviral portion (Table 2 and Fig. 6). Moreover, in the specific case of the duplicated sequence in locus 6p21.33, the presence of a low T value could possibly be biased by the fact that these sequences are located within an important genic region, presenting an overall lower substitution rate, and, for sequence 6p21.33b, the fact that has been recently created by a large gene duplication. It is worth to note that the apparent loss of both 6p21.33 proviral copies in different evolutionarily intermediate primates species, as already reported [32], is another confounding factor for the accurate T estimation of these elements.

Finally, it is interesting to note that HML10 type II sequences are older than HML10 type I insertions, showing an average estimated time of integration of 35,5 mya ago with respect to a medium age of 25, 9 mya calculated for type I elements.

### Comparative identification of orthologous insertions in non-human primates

Most HERVs entered into the primates lineages between 10 and 50 mya, during primates evolutionarily speciation. The most ancient HERV-K HML group, the HML-5 one, has been estimated to have integrated before the separation of New and Old World Monkeys, occurred about 43 mya, while the other HMLs appeared later on in several subsequent waves of colonization of the *Catarrhini* parvorder only (Old World Monkeys and Hominoids). Hence, in order to gain more details on the HML10 diffusion in the various primate species, we searched the HML10 sequences orthologous to each provirus retrieved in the human genome in the genome assemblies of one New World Monkey (Marmoset; *Platyrrhini* parvorder), one Old World Monkey (Rhesus macaque; *Catarrhini* parvorder) and 4 Hominoids (Gibbon, Orangutan, Gorilla and Chimpanzee; *Catarrhini* parvorder). As shown in Table 3, six of the nine HML10 proviruses found in the human genome have corresponding orthologous sequences in all the analyzed *Catarrhini* species, from Chimpanzee to Rhesus, confirming an approximate main period of HML10 group diffusion between 43 and 30 mya. 1p22.2 partial provirus is also present from human to Rhesus, but its orthologous insertion in the Gorilla genome is missing, possibly due to a deletion event. With regards to the provirus integrated in locus 6p21.33, the two identical copies are localized in the human complement C4A and C4B genes, known to reside on duplicated segments of DNA. In particular, the C4 genes of some *Catarrhini* primates exhibit a long/short dichotomous size variation due to the presence/absence of these HML10 integrations, while chimpanzee and gorilla only contain short C4 genes [19, 32]. In line with this, 6p21.33a and 6p21.33b orthologous HML10 insertions were localized in Rhesus and Orangutan genome sequences, respectively, but are absent in the other analyzed species (Table 3). Finally, the orthologous HML10 provirus in locus Yq11.221 could be localized in Chimpanzee genome only, because no comparative information are available for the Y chromosome of the other primate species (Table 3).

**Table 3:** HML10 sequences orthologous loci in non-human primates genome

Human locus	Chimpanzee	Gorilla	Orangutan	Gibbon	Rhesus	Marmoset
1p36.13 (−)	1:19,897,252–19,903,183 (−)	1:20,573,241–20,579,060 (−)	1:210,407,411–210,413,307 (+)	24:19,115,921–19,117,286 (−)	1:22,729,037–22,740,752 (−)	x
1p22.2 (−)	1:89,883,243–89,885,583(−)	x	1:139,752,930–139,755,294 (+)	12:87,503,425–87,505,758	1:92,543,319–92,545,983 (−)	x
1q22 (−)	1:133,941,236–133,948,931 (−)	1:134,686,645–134,687,185 (−)	1:95,817,622–95,818,162 (+)	assembly gap	1:134,772,475–134,779,343 (−)	x
6p22.1 (+)	6:27,446,871–27,456,058 (+)	6:28,001,913–28,010,233 (+)	6:28,071,758–28,078,582 (+)	1a:72,438,487–72,447,474 (+)	4:27,112,448–27,121,339 (+)	x
6p21.33a (−)	x	x	x	x	4:32,223,558–32,230,572 (−)	x
6p21.33b (−)	x	x	6:32,500,019–32,506,424 (−)	x	x	x
6q22.31 (−)	6:123,707,066–123,714,005 (−)	6:122,872,935–122,879,489 (−)	6:125,032,218–125,039,364 (−)	3:109,711,272–109,718,216 (−)	4:143,675,558–143,676,403 (−)	x
19p13.2 (−)	19:7,923,717–7,929,241 (−)	19:8,020,313–8,024,861 (−)	19:7,962,003–7,966,295 (−)	10:66,445,268–66,447,647 (+)	19:8,140,869–8,144,331 (+)	x
19q13.41 (−)	19:57,389,749–57,395,370 (−)	19:49,869,509–49,875,109 (−)	19:53,964,824–53,970,559 (−)	10:72,725,038–72,730,734 (−)	19:58,261,760–58,267,798 (−)	x
Yq11.221 (−)	Y:20,496,417–20,503,728 (−)	–	–	–	–	–

For each human HML10 locus (for precise start and end positions, see Table 1), chromosome coordinates and strand of orthologous loci are given for the other regarded non-human *Catarrhini* primate reference genome sequences. Apparent absence of a HML10 sequence in the orthologous genome position is indicated by “x”. Regarding the HML10 locus on the human chromosome Y, comparative information is available for chimpanzee genome sequence only (see main text)

In addition to the non-human primates HML10 sequences orthologous to human loci, we wanted also to assess whether the group period of proliferation activity could have also determined species-specific insertions outside of the human evolutionary lineage. Thus, we performed BLAT searches in the above mentioned non-human primates genome sequences using the HML10 group LTR14-HERVKC4-LTR14 RepBase sequence [34] from Dfam database [38] as a query. The analysis showed that no additional species-specific HML10 integrations are present in Chimpanzee, Gorilla, Orangutan and Rhesus genome sequences (data not shown), while a HML10 provirus apparently lacking orthologous loci in the other primate species was found in Gibbon assembly chr5:62,078,165–62,086,762. This provirus was in part recognized as HML9 sequence based on RepeatMasker annotation track, but its inclusion in a NJ phylogenetic tree with all the 10 HML groups reference sequences confirmed its belonging to the HML10 group (data not shown).

### Retroviral features analysis

**Fig. 7:**
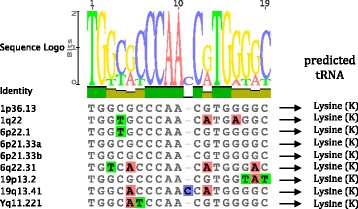
HML10 proviruses PBS analyses. Nucleotide alignment of the PBS sequences identified in the HML10 proviruses. In the upper part, a logo represents the general HML10 PBS consensus sequence: for each nucleotide, the letter height is proportional to the degree of conservation among HML10 members. As indicated, all the HML10 PBS sequences are predicted to recognize a Lysine (K) tRNA

Beside these major determinants, the various HERV genera share some specific features, which are also valuable for taxonomic purposes [49]. Particularly, it is known that Class II Betaretrovirus-like HERVs, including the HERV-K HML1–10 groups, commonly present a PBS sequence putatively recognizing a Lysine (K) tRNA. The human tRNA supposed to prime the retrotranscription process, in fact, has been used for a long time for HERV nomenclature and, even if now it is considered poorly reliable for taxonomic classification, it remains a characteristic feature of the different HERV groups. Among the nine HML10 proviruses analyzed, eight conserve a PBS sequence, while locus 1p22.2 provirus is defective for a big 5′ retroviral portion and lacks 5’LTR and *gag* gene. As expected, when present, the PBS sequence is located 3 residues downstream the 5’LTR and is 18 nucleotide in length, except for 19q13.41 provirus that has a single nucleotide insertion between residues 10 and 11 (Fig. 7). All the analyzed PBS were predicted to recognize a Lysine tRNA and show a conserved nucleotide composition, as indicated in the logo generated from the PBS sequences alignment (Fig. 7).

Other common features of Class II Betaretrovirus-like HERV groups are i) a Pro C-terminal G-patch motif, ii) a Pro N-terminal dUTPase, and iii) two Gag NC Zinc finger motifs [3, 49]. In the case of the HML10 sequences, however, these features are not present due to the absence of the harboring retroviral genome portions. As described, in fact, all HML10 proviruses lack the entire *pro* gene and, with the exception of locus 1p36.13, the *gag* NC portion (Fig. 3). However, the analysis of HML10 locus 1p36.13 revealed also in this provirus the partial deletion of the gene 3′ terminal portion, i.e. the one normally including both the Zinc finger motifs.

Finally, the HML10 group is known to be biased for the Adenine (A) content, showing around the 34% of A and only the 17% of Guanine (G) nucleotides in the canonical sequences [3]. Such G to A hypermutation could be due to host RNA editing systems, as commonly observed with APOBEC3G enzymes in *Lentiviruses* [50]. The analysis of our complete dataset nucleotide frequencies confirmed a bias for A, showing in average a 33% of A (maximum = 36%, minimum = 31%, standard deviation = 2) and a 18% of G (maximum = 21%, minimum = 15%, standard deviation = 2). In addition to this skewed purine composition, we observed a weak bias in pyrimidine amount, with 28% of Thymine (T) (maximum = 28%, minimum = 27%, standard deviation = 1) and 21% of Cytosine (C) (maximum = 22%, minimum = 19%, standard deviation = 1).

### Phylogenetic analyses

**Fig. 8:**
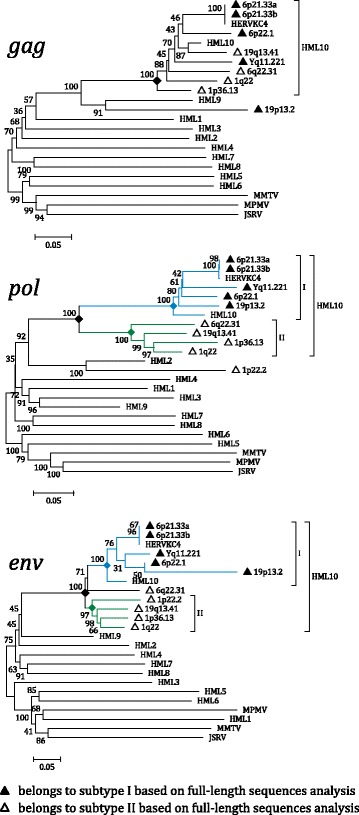
Phylogenetic analysis of the HML10 sequences *gag*, *pol* and env genes with other endogenous and exogenous Betaretroviruses. The main HML10 phylogenetic group is indicated. The two intragroup clusters (I and II), when present, are also annotated and depicted with blue and green lines, respectively. In the absence of clear cluster division, the belonging of each element to the two subgroups is indicated based on the full-length proviruses phylogenetic analysis (Fig. 2). Evolutionary relationships were inferred by using the Neighbor Joining method and the Kimura-2-parameter model. The resulting phylogeny was tested by using the Bootstrap method with 1000 replicates. Length of branches indicates the number of substitutions per site

To gain more insights into the HML10 group phylogeny, we analyzed all identified HML10 proviruses using the nucleotide sequences of *gag*, *pol* and *env* genes to generate NJ trees, including also the reference sequences of all Dfam HERV-K groups (HML-1 to 10) and of some representative exogenous Betaretroviruses (MMTV, MPMV and JSRV) (see Methods) (Fig. 8). The presence of two types of HML10 proviruses, was confirmed in the NJ trees of both *pol* and *env* genes, but not in the *gag* gene (Fig. 8), in agreement with the HML10 individual loci structural characterization, which already pointed out that the major differences between type I and type II elements are located in the *pol* RNase H and IN portions and in the *env* 5′ region. More in details, the *gag* gene phylogenetic analysis revealed that all HML10 sequences group together with 100 bootstrap support, except for 19p13.2 provirus, which was related instead to the HML9 reference sequence. Due to the fact that this HML10 provirus has been inserted as a secondary integration within a pre-existing HML9 proviral sequence, a part of the flanking HML9 element could have been erroneously associated to the encompassed HML10 element. To assess this possibility, we analyzed 19p13.2 HML10 with respect to both HML10 and HML9 Dfam references with Recco software [51], detecting eventual recombination events among aligned sequences (data not shown). Indeed, an internal portion of the 19p13.2 provirus (from nucleotide 755 to nucleotide 1384, 15% of the total length) is effectively more similar to HML9 reference, being albeit included in a “true” HML10 proviral sequence (nt 1–754 and 1285–4986, 85% of the total length) and suggesting the previous occurrence of a recombination event involving the gag gene and leading to a HML10 mosaic form (data not shown).

Differently, in *pol* tree the phylogenetic clusters of type I and II proviruses were supported by the maximum bootstrap value (100), including all the respective proviruses as already classified based on the full length nucleotide sequence, except for locus 1p22.2. The latter *pol* sequence, similarly to what observed for locus 19p13.2 *gag* gene, showed instead higher similarity to the HML2 group reference sequence. The same type I and II phylogenetic clusters have been observed in *env* gene phylogenetic analysis, showing also in this case a high bootstrap support (100 and 98, respectively). In this tree, subtype II sequence in locus 6q22.31 showed an intermediate position, sharing some high similarities with type I cluster also.

For completeness, we analyzed the phylogeny of the HML10 proviral 5′ and 3’LTR also, including the LTR references for HML1 to 10 groups and for the exogenous Betaretroviruses MMTV, MPMV and JSRV. As expected, all the HML10 proviruses 5′ and 3’LTR sequences grouped together with the group reference LTR14, supported by a 100 bootstrap value (Additional file 1: Figure S1). Within this phylogenetic group, both LTRs of the same proviral element were generally coupled with bootstrap values ranging from 91 to 100, but no clusters dividing the LTRs of type I and type II HML proviruses were observed, confirming an overall common LTR sequence for both subgroups (Additional file 1: Figure S1).

## Discussion

Initially identified due to the presence of an integrated proviral sequences in the human C4 gene [32], the HML10 group expression has been proposed to affect a number of biological processes. The HERV-K(C4) prototype sequence is, in fact, normally expressed in various human cells, almost exclusively producing antisense transcripts [18, 21] that have been hypothesize to act as i) regulators of homologous genes expression through antisense inhibition, ii) possible defense mechanism against exogenous infections, iii) potential contributor to autoimmune diseases involving the complement components [21]. Recently, some HML10 proviruses, other than HERV-K(C4) and originally reported by Vargiu et al. [3], have been investigated for their promoter capacity and expression, further supporting their possible role as antisense regulators of host genes [27]. This is of particular interest, considering that most HML10 elements are located within human introns in antisense orientation, and many of them, in addition to the well studied HERV-K(C4) insertions, can potentially influence host functions. Interestingly, the antisense expression of HML10 provirus in locus 1q22 downregulated the encompassing gene DAP3 in cell culture, leading to an apoptotic-resistant cell phenotype [27]. These findings, together with the reported generic group expression in various tumor cell lines, could suggest a contribution of some HML10 loci to human malignancies, potentially through to the loss of apoptosis cell control. Overall, while these findings made the HML10 group one of the most interesting HERV groups, the lack of the complete identification of the HML10 integrations and the lack of a comprehensive investigation of the single HML10 loci impeded the assessment of their specific contribution to human transcriptome and to human pathogenesis [52].

In the present work, we completed the identification of the HML10 proviruses, reporting for the first time an additional HML10 sequence in locus 1p22.2. The latter, even if characterized by a defective structure, being 2337 nucleotides in length and showing the *pol* and *env* genes only, constitutes a partial but “true” HML10 provirus based on structural and phylogenetic analyses. Hence, given the HML10 proviruses reported in our previous study [3], there are nine HML10 sequences in the human genome. In addition, we analyzed and characterized in great detail the structure, phylogeny and estimated period of diffusion of these ten HML10 proviruses providing, to our knowledge, the most complete representation of the HML10 group up to date. The chromosomal distribution of these proviruses and the HML10 solitary LTR relics revealed a non-random integration pattern, showing clusters of sequences with a number of integration higher than expected, especially in chromosomes 6, 9, 19, X and Y. This bias, in the case of gene-rich chromosomes such as 17 and 19 ones, is probably linked to the strong preference of HML10 elements to be inserted in proximity or within human gene introns [3, 27], while for the Y chromosome, showing a lower recombination rate, it could be linked to a greater rate of HERV fixation [37]. The phylogenetic analysis of the full length proviral nucleotide sequences revealed the presence of two well supported clusters, identified here as type I and II and including 4 and 5 members, respectively, and further confirmed by the phylogenetic analysis of both *pol* and *env* genes. Interestingly, the structural analysis of such regions showed that both types of HML10 sequences have some specific domains, being present in all the same-type members but not found in the correspondent portion of the other-type sequences. In the case of type I sequences, we found that the A/T-rich stretch previously reported between the *pol* and *env* genes of HERV-K(C4) provirus [14] is present also in the other 3 type I elements. Similar A/T-rich regions have been reported also in other HERV LTRs [53, 54] as well as in the *env* gene of a HML2 provirus in locus 5q33.2 [42], but the function of such portion in these sequences as well as in HML10 type I elements is still unknown. In the case of type II HML10 elements, the portion corresponding to type I intergenic A/T-rich stretch presents instead putative functional domains of Pol and Env proteins not found in type I proviruses, such as the RNase H 5′ portion, the IN core and DNA binding domains and, of further note, an Env Rec domain, whose presence has been confirmed also through the phylogenetic analysis of the five type II HML10 proviruses Rec puteins. Until now, Rec was considered to be exclusive of a subset of HERV-K(HML2) sequences [40–42]. HML2 Rec has been shown to be expressed in a wide range of tissues [55], interacting with a number of cellular proteins relevant for host physiological functions [56–59], and is currently highly investigated for its oncogenic potential (as reviewed in [60, 61]). Thus, the expression of a Rec analogue in HML10 sequences could contribute to human physiopathology and surely deserves to be further investigated, given that two of the five characterized HML10 Rec puteins did not harbor any premature stop codon or frameshift and presented a putatively functional NES. Other interesting structural peculiarities of HML10 group are the absence of *pro* gene and the presence of a shorter *gag* gene lacking the nucleocapsid portion, that was found only in 1p36.13 type II provirus. Apart from the possibility of an occasional loss of *pro* due to post-insertional mutations and deletions, such gene is usually present in HERV sequences, being often the most intact ORF [3]. Thus, to our knowledge, HML10 is the first HERV group systematically lacking the *pro* gene. While unlikely, it is hence possible to speculate that its original exogenous retroviruses could have evolved alternative mechanisms for protein cleavage, as observed for the coopted HERV-W Syncytin-1 Env, in which a peculiar four amino acids deletion made the protein constitutively fusogenic even in the absence of a functional viral Protease [62]. While such diffuse defective structure in *pro* and *gag* genes implied the absence of the relative Betaretroviruses characteristic features (Pro G-patch and dUTPase, Gag Zinc fingers), 8/9 HML10 sequences maintained the originally reported PBS sequence recognizing a K tRNA. Also the previously reported purine bias [3] was confirmed, showing an A frequency average of about 33%, and an unreported weak bias in pyrimidines amount, with an increase in T percentage (28%). The G to A bias could be explained by the action of host RNA APOBEC editing enzymes, as observed for HIV-1 [50] and HERV-K(HML2) [63] sequences, while the C to T hypermutation could be due to DNA methyltransferase methylation of CG dinucleotides, followed by the spontaneous deamination of methyl-C to T, as a potential silencing mechanism of retroelements. The time of integration estimation, performed for each HML10 sequence with a multiple and more reliable approach suggested that HML10 elements have been acquired by the primate lineages between 40 and 20 mya and mostly found in all the analyzed *Catarrhini* primates, but not in *Platyrrhini* species. This estimation was further corroborated by the identification of each human locus orthologous HML10 insertion in the genome assembly of 5 *Catarrhini* non-human primates species, providing the first comparative map of the group. This analysis also revealed a HML10 species-specific insertion in Gibbon chromosome 5, hence acquired after the evolutionary separation from subsequent species, i.e. less than 20 mya.

## Conclusions

Besides the well studied HERV-K(C4) proviruses, also other HML10 sequences can be involved in the antisense control of homologous gene expression, possibly contributing to immune regulation and antiviral defense, as well as having a role in cancer development and autoimmunity. The present exhaustive characterization of all the HML10 sequences integrated in the human genome is thus the needed comprehensive background that is essential to assess the physio-pathological effects of HML10 expression.

## Methods

### HML10 sequences localization in human and non-human primates genomes

The HML10 sequences integrated in human genome assembly GRCh37/hg19 were identified based on the previous analysis of the latter with RetroTector software [3] combined with a UCSC Genome Browser [64, 65] BLAT search using the RepBase Update [34] assembled reference LTR14-HERVKC4-LTR14 as a query.

The HML10 loci orthologous to each human sequence have been identified through the comparative localization of the harboring genomic region for the following *Catarrhini* primate genome assemblies in UCSC Genome Browser:

- Chimpanzee (*Pan troglodytes*, assembly Feb. 2011 - CSAC 2.1.4/panTro4)
- Gorilla (*Gorilla gorilla gorilla*, assembly May 2011 - gorGor3.1/gorGor3)
- Orangutan (*Pongo pygmaeus abelii*, assembly July 2007 - WUGSC 2.0.2/ponAbe2)
- Gibbon (*Nomascus Leucogenys*, assembly Oct. 2012 - GGSC Nleu3.0/nomLeu3)
- Rhesus (*Macaca mulatta*, assembly Oct. 2010 - BGI CR_1.0/rheMac3)

while the search in Marmoset (*Platyrrhini* parvorder) genome sequence (*Callithrix jaccus*, assembly March 2009 - WUGSC 3.2/calJac3) gave negative results.

The eventual HML10 species specific insertion lacking an ortholog in humans have been searched in the same non human primates genome sequences through a UCSC Genome Browser [64, 65] BLAT search using the RepBase Update [34] assembled reference LTR14-HERVKC4-LTR14 as a query.

### Analysis of HML10 chromosomal distribution

In order to estimate the expected number of integration events, each human chromosome length has been multiplied for the total number of HML10 insertions, including both proviruses and solitary LTR relics, and the obtained value has been divided for the total length of the human genome sequence. The number obtained, representing the expected proportion of HML10 insertion for each chromosome based on a random distribution principle, has been then compared to the actual amount of HML10 sequences.

### HML10 proviral sequences alignment

Pairwise and multiple alignments of HML10 proviral nucleotide sequences were generated with Geneious bioinformatics software platform, version 8.1.4 [66] using MAFFT algorithm G-INS-i [67] with default parameters.

Pairwise and multiple alignments of HML10 puteins amino acid sequences were generated with Geneious bioinformatics software platform, version 8.1.4 [66] using MAFFT algorithm G-INS-i [67] with default parameters, after the bioinformatics translation of the correspondent gene portion.

All alignments have been visually inspected and, if necessary, manually corrected before further structural and phylogenetic analyses. The multiple alignment of the 9 HML10 proviral sequences with respect to LTR14-HERV-K(C4)-LTR14 reference is provided in fasta format as Additional file 2

### Phylogenetic analyses

All phylogenetic trees were built from manually optimized multiple alignments generated by Geneious (see above) using Mega Software, version 6 [68] and NJ statistical method. Nucleotide and amino acid sequences NJ trees were built using the p-distance model and applying pairwise deletion option. Phylogenies were tested by the bootstrap method with 1000 replicates.

Beside HML10 proviral sequences, the trees included also the following reference sequences, as representative for endogenous and exogenous Betaretroviruses:

- HML10 prototype HERV-K(C4) RepBase [34] assembled nucleotide sequence (LTR14-HERVKC4-LTR14)
- HML1–10 Dfam [38] assembled nucleotide sequences: HML1 (LTR14A-HERVK14-LTR14A), HML2 (LTR5-HERVK-LTR5), HML3 (MER9B-HERVK9-MER9B), HML4 (LTR13-HERVK13-LTR13), HML5 (LTR22A-HERVK22-LTR22A), HML6 (LTR3-HERVK3-LTR3), HML7 (MER11D-HERVK11D-MER11D), HML8 (MER11A-HERVK11-MER11A), HML9 (LTR14C-HERVK14C-LTR14C) and HML10 (LTR14-HERVKC4-LTR14)
- MMTV nucleotide sequence (GenBank accession number: NC_001503.1)
- MPMV nucleotide sequence (GenBank accession number: NC_001550.1)
- JSRV nucleotide sequence(GenBank accession number: NC_001494.1)
- GenBank representative Rec proteins and their exogenous analogues amino acid sequences: HERV-K HML2 (Q69383.1, P61573.1, P61576.1, P61575.1, P61574.1, P61572.1, P61578.1, P61579.1, P61571.1), HIV-1 Rev. (NP_057854), HTLV-1 Rex (NP_057863), STLV-1 Rex (NP_056908)

### Structural analyses

The nucleotide sequence of each HML10 provirus has been aligned to the HML10 prototype HERV-K(C4) RepBase [34] assembled reference (LTR14-HERVKC4-LTR14) and all insertions and deletions ≥1 nucleotide as well as the main structural and regulatory features have been annotated in a graphical representation of the multiple alignment. The prediction of functionally relevant domains has been performed with the NCBI tool for conserved domains search [39] (https://www.ncbi.nlm.nih.gov/Structure/cdd/wrpsb.cgi)

### PBS type and Betaretroviral features characterization

The PBS nucleotide sequence of each HML10 provirus has been aligned and compared with a library of 1171 known HERV PBS [3] to assign the most probably recognized tRNA. The general conservation of the PBS sequence among the HML10 proviruses has been represented by a logo generated at http://weblogo.berkeley.edu/logo.cgi [69] from the nucleotide alignment of all the HML10 PBS sequences.

The features known to be associated to Betaretroviruses, i.e. a Pro C-terminal G-patch motif (GYx2GxGLGx4GxnG), a Pro N-terminal dUTPase (DSDYxGEIQ), and two Gag NC Zinc finger motifs (CX2CX4HX4C) [3] were manually searched after the bioinformatics translation of the harboring genes (when present) in all the three possible reading frames with Geneious bioinformatics software platform, version 8.1.4 [66].

In order to individuate any bias in the HML10 sequences nucleotide composition, the relative frequencies of each nucleotide in the individual proviruses has been estimated by Geneious bioinformatics software platform, version 8.1.4 [66], after the manual removal of any eventual secondary integration. The final value for each nucleotide has been expressed as the average value obtained in the single HML10 proviruses.

### Time of integration estimation

The time of integration of each HML10 provirus was estimated using a multiple approach of calculation, based on the percentage of divergent nucleotides (D%) between i) the two LTRs of each sequence, ii) each LTR of each sequence and a HML10 LTR consensus generated from our dataset alignment, and iii) the *gag*, *pol* and *env* genes of each sequence and a HML10 *gag*, *pol* and *env* consensus generated from our dataset alignment. Regarding *pol* and *env* genes, the nucleotides region showing high divergence between the two types of sequences were excluded, considering only the portions sharing a general identity comparable to the rest of the proviral structure (nucleotides 1277–2571 and 4103–5810 in LTR14-HERVKC4-LTR14 reference assembled reference, respectively). In particular, the pairwise D% between aligned nucleotide sequences was estimated, after removal of hypermutating CpG dinucleotides, by MEGA Software, version 6 [68], through a p-distance model with the pairwise deletion option applied**.** Variance was estimated by Neighbor Joining method with 1000 bootstrap replicates.

The estimated time of integration (T) was obtained according to the relation:

$$ \mathrm{T}=\mathrm{D}\%/0,2\% $$

where 0.2% correspond to the neutral substitution rate acting on the human genome (percentage of mutation per nucleotide per million years). With regards to the D% between the two LTRs of the same provirus, which are known to be identical at time of integration, the T obtained was further divided by a factor of 2, considering that each LTR accumulates mutation independently.

For each HML10 provirus, the final T was expressed as the mean of the values obtained through the three approaches of D% calculation, after the exclusion of values with standard deviation >20%.

## Additional files

Additional file 1: Figure S1. Phylogenetic analysis of the HML10 sequences 5'- and 3'LTRs with other endogenous and exogenous Betaretroviruses. The main HML10 phylogenetic group is indicated. In the absence of clear cluster division, the belonging of each element to the two subgroups is indicated based on the full-length proviruses phylogenetic analysis (Fig. 2). Evolutionary relationships were inferred by using the Neighbor Joining method and the Kimura-2-parameter model. The resulting phylogeny was tested by using the Bootstrap method with 1000 replicates. Length of branches indicates the number of substitutions per site. (PDF 12 kb)

Additional file 2: HML10 multiple alignment. FASTA multiple alignment of the 9 HML10 proviral sequences with respect to LTR14-HERV-K(C4)-LTR14 RepBase reference. (FASTA 149 kb)

## Abbreviations

C4: fourth component of human complement gene
CYP21: steroid 21-hydroxylase
D: Divergence
DAP3: Death-associated protein 3
HERV: Human Endogenous Retroviruses
HIV-1: Human Immunodeficiency Virus 1
HML: Human MMTV-like
HTLV-1: Human T Lymphotropic Virus 1
IN: Integrase
JSRV: Jaagsiekte Sheep Retrovirus
LTR: Long Terminal Repeats
MHC: major histocompatibility complex
MMTV: Mouse Mammary Tumor Virus
MPMV: Mason-Pfizer Monkey Virus
mya: million years ago
NJ: Neighbor Joining
O.C.A.: Oldest Common Ancestor.
ORF: Open Reading Frame
PBS: Primer Binding Site
PPT: polypurine tract
puteins: putative proteins
RDDP: RNA Dependent DNA Polymerase
ReTe: RetroTector software
RNase H: Ribonuclease H
RP: serine/threonine nuclear protein kinase
RT: Reverse Transcriptase
STLV-1: Simian T Lymphotropic Virus 1
T: Time of integration
TNX: Tenascin extracellular matrix protein
