# Supplementary figures and images for: Identification of a novel HERV-K(HML10): comprehensive characterization and comparative analysis in non-human primates provide insights about HML10 proviruses structure and diffusion
Source: Mob DNA. 2017 Nov 2;8:15. doi: 10.1186/s13100-017-0099-7 (PMC5667498; doi:10.1186/s13100-017-0099-7)

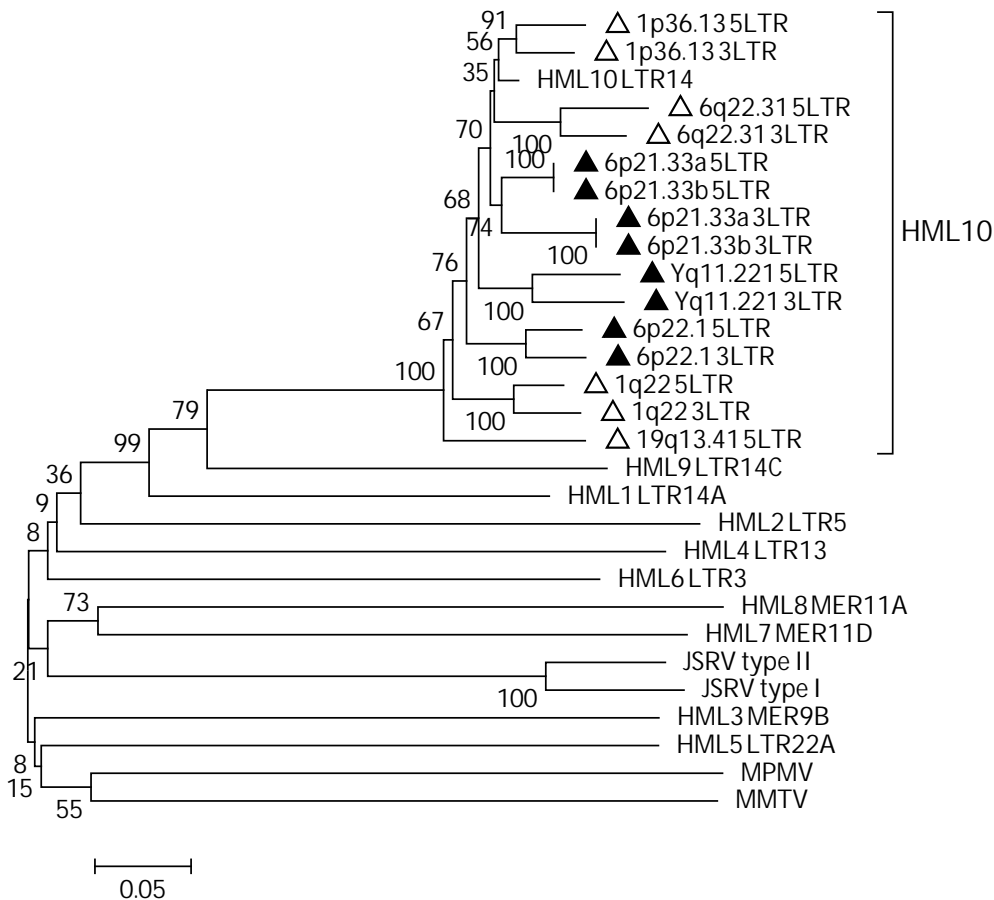

Supplement: Supplementary file 1 — Phylogenetic analysis of the HML10 sequences 5'- and 3'LTRs with other endogenous and exogenous Betaretroviruses. The main HML10 phylogenetic group is indicated. In the absence of clear cluster division, the belonging of each element to the two subgroups is indicated based on the full-length proviruses phylogenetic analysis (Fig. 2). Evolutionary relationships were inferred by using the Neighbor Joining method and the Kimura-2-parameter model. The resulting phylogeny was tested by using the Bootstrap method with 1000 replicates. Length of branches indicates the number of substitutions per site. (PDF 12 kb) [file 13100_2017_99_MOESM1_ESM.pdf]
